# Dermoscopy of the iris: identification of Lisch nodules and contribution to the diagnosis of Neurofibromatosis type 1^☆^^☆☆^

**DOI:** 10.1016/j.abd.2020.09.007

**Published:** 2021-05-21

**Authors:** Luciana Pavan Antoniolli, Laura de Mattos Milman, Renan Rangel Bonamigo

**Affiliations:** Dermatology Service, Hospital de Clínicas de Porto Alegre, Universidade Federal do Rio Grande do Sul, Porto Alegre, RS, Brazil

**Keywords:** Dermoscopy, Neurocutaneous syndromes, Neurofibromatoses, Neurofibromatosis 1

## Abstract

Neurofibromatosis is a common genodermatosis, whose diagnosis often involves the participation of a dermatologist. A case of a 38-year-old female patient with four *café-au-lait* macules and eleven neurofibromas on clinical examination is presented. Dermoscopy allowed the identification of Lisch nodules in the iris, bilaterally. The combination of these findings allowed the diagnosis of neurofibromatosis type 1, according to NIH criteria. Lisch nodules are melanocytic hamartomas of the iris, which must be evaluated through a visual augmentation method, usually employed in ophthalmology. Alternatively, dermoscopy can be used and contribute to the early diagnosis of neurofibromatosis type 1.

## Case report

A 38-year-old female patient was referred to the Dermatology Service for the evaluation of nodular lesions present since adolescence, with progressive growth. On physical examination, she had ten soft, normochromic nodules, distributed on the trunk and limbs, compatible with neurofibromas. She also had a lesion measuring 10 cm in diameter, on the back of the left thigh, compatible with a plexiform neurofibroma, and four *café-au-lait* macules, measuring 1 to 3 cm in diameter, distributed on the trunk. She had no axillary or inguinal freckling. She denied a history of visual or bone alterations. She reported a history of cognitive impairment, which had not been objectively assessed, and a paternal family history of similar skin lesions, without a diagnosis and no possibility of objective assessment at that time.

**Figure 1:**
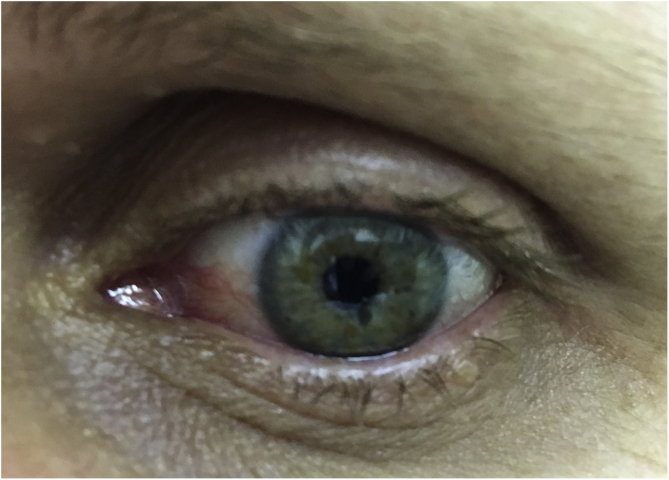
To the naked eye: Possible brownish-yellow spots on the iris.

**Figure 2:**
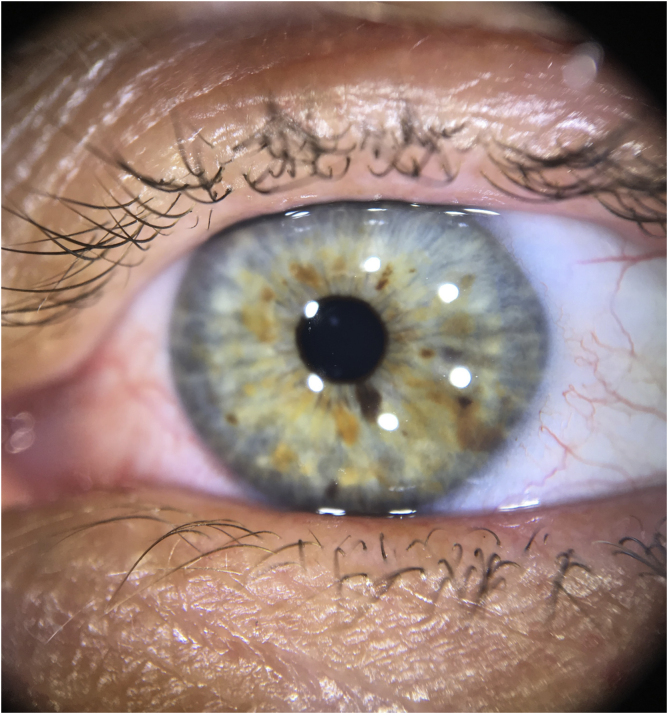
Dermoscopy with polarized light: Lisch nodules on the iris (brownish-yellow lesions of varying hues, with wide distribution).

At this time, the patient had several findings suggestive of neurofibromatosis type 1; however, she still did not meet the diagnostic criteria of the National Institutes of Health (NIH) (Table 1).^1^ The authors then observed that the patient had light-colored irises, with some brown spots (Fig. 1). Iris dermoscopy was performed, in which brownish-yellow nodules were observed bilaterally, compatible with Lisch nodules (Fig. 2). The combination of these clinical findings allowed the diagnosis of neurofibromatosis type 1, according to the NIH criteria. The patient was referred for multidisciplinary evaluation with the Genetics, Neurology, Ophthalmology, and Surgical teams.

**Table 1:** Diagnostic criteria for neurofibromatosis type 1^a^ (National Institutes of Health, 1990)

1. Six or more *café au lait* macules: >0.5 cm of extension in children or >1.5 cm in adult patients
2. Two or more cutaneous/subcutaneous neurofibromas or one plexiform neurofibroma
3. Axillary or groin freckling
4. Optic pathway glioma
5. Two or more Lisch nodules (pigmented iris hamartomas)
6. A characteristic bone lesion, such as sphenoid bone dysplasia or thinning of the long bones of the cortex, with or without pseudoarthrosis
7. First degree relative with neurofibromatosis type 1

a Two or more of the criteria are required for diagnosis.

## Discussion

The diagnosis of neurofibromatosis usually requires a multidisciplinary team, in which the role of the dermatologist is essential. It is based on the NIH clinical criteria and generally does not require genetic testing. These criteria are highly sensitive and specific, except in very young children.^1^, ^2^ Early diagnosis of this genodermatosis is important for the monitoring of lesions and preventing complications.

Lisch nodules are well-defined melanocytic iris hamartomas, with a dome appearance, light brown color, and do not cause any visual alterations.^3^, ^4^ They usually appear between two and six years of age and are present in more than 90% of adult patients with neurofibromatosis.^3^ Eventually, they may be visible to the naked eye, especially if the iris is light in color and the nodules are large and numerous, but evaluation with visual enlargement is recommended. Traditionally, ophthalmic examination with a slit lamp is performed; recently, the possibility of detection through dermoscopy has been described, including in brown eyes.^4^, ^5^

The observation of Lisch nodules can be especially useful in young children with multiple *café-au-lait* macules and no family history of neurofibromatosis, as they appear before the neurofibromas.^4^ Evaluation by an ophthalmologist is indicated to differentiate them from granulomatous iritis and from iris mammillations, nevi, and iris melanoma; as well as for the diagnosis of tumors associated with neurofibromatosis, which can cause visual alterations, such as optic pathway gliomas.^1^, ^4^ Additionally, the presence of Lisch nodules is characteristic of neurofibromatosis type 1, being absent in neurofibromatosis type 2.^1^

This is the report of an adult patient with neurofibromas and *café-au-lait* macules, but without a previous diagnosis, for which the observation of Lisch nodules through dermoscopy was important for the diagnosis of neurofibromatosis type 1. Therefore, the authors highlight that dermoscopy can be an auxiliary tool for the dermatologist to assess cutaneous and the ocular lesions of this genodermatosis.

## Financial support

None declared.

## Authors’ contributions

Luciana Pavan Antoniolli: Approval of the final version of the manuscript; design and planning of the study; drafting and editing of the manuscript; intellectual participation in the propaedeutic and/or therapeutic conduct of the studied cases; critical review of the literature.

Laura de Mattos Milman: Approval of the final version of the manuscript; design and planning of the study; intellectual participation in propaedeutic and/or therapeutic conduct of studied cases; critical review of the manuscript.

Renan Rangel Bonamigo: Approval of the final version of the manuscript; design and planning of the study; intellectual participation in propaedeutic and/or therapeutic conduct of studied cases; critical review of the manuscript.

## Conflicts of interest

None declared.
